# In vitro evaluation of human enamel remineralization after treatment with Ginger, Ashwaganda and Maca herbal dentifrices versus commercially available fluoride containing dentifrice

**DOI:** 10.1038/s41405-025-00298-9

**Published:** 2025-03-03

**Authors:** Ghada Ahmed Elzayat, Fagr Hassan Elmergawy, Aya Abd ElFattah Mohammed Nemt Allah

**Affiliations:** 1 Lecturer of Conservative dentistry, Faculty of Dentistry, Egyptian Russian University, Cairo, Egypt; 2 Lecturer of Dental Biomaterials, Faculty of Dentistry, October university for modern sciences and arts (MSA), Giza, Egypt; 3 Lecturer of Conservative dentistry, Faculty of Dentistry, Egyptian Russian University, Cairo, Egypt

**Keywords:** Tooth brushing, Oral diseases

## Abstract

**Background:**

Compare the remineralization efficiency of Ginger, Ashwaghanda and Maca dentifrices versus commercially fluoride containing dentifrice.

**Methods:**

Ginger, Ashwaghanda and Maca extracts were prepared by solvent extraction methodology and were characterized using transmission electron microscope, dynamic light scattering, and inductively coupled plasma optical emission spectrometer. The pH of the dentifrices was evaluated by pH meter. Eighty teeth were collected and divided into five groups according to the treatment protocol. Enamel morphology was carried out by scanning electron microscope with energy dispersive X-Ray spectroscopy for the analysis of calcium, phosphorus, Ca/P ratio and carbon. Surface microhardness was evaluated by Vickers micro-hardness tester. Data were analyzed using One-way ANOVA followed by Tukey’s post hoc test (*p* ≤ 0.05).

**Results:**

Characterization results showed the highest calcium, phosphorus and fluoride ion release were associated to Maca, Ashwaganda and Ginger respectively. The pH results revealed that Ginger dentifrice exhibited the most alkaline pH, whereas Ashwaganda dentifrice exhibited the most acidic pH. Morphological analysis revealed that Ashwaganda showed lower remineralization ability compared to the other treated groups. Maca showed significant higher Ca/P ratio compared to other groups (*p* < 0.001) and Ginger showed significant higher surface microhardness recovery compared to Ashwaganda (*p* < 0.001).

**Conclusion:**

Ginger and Maca are promising remineralizing agents.

## Introduction

Human teeth are continuously subjected to dynamic cyclic periods of demineralization and remineralization processes. Where, demineralization includes loss of minerals from the hydroxyapatite (HA) crystal lattice in tooth structures due to decrease in pH conditions. Whereas, remineralization restores the minerals again to the HA crystals as natural repair mechanism [1]. Regular use of oral hygiene products is considered one of the main strategies to enhance tooth remineralization. The use of tooth brushing with fluoridated dentifrices has been the most effective non-professional oral hygiene method to prevent and treat early demineralized tooth lesions owing to the ability of fluoride to promote the formation of fluorapatite crystals which are more resistant to demineralization. Fluoridated dentifrices can also maintain high levels of fluoride in the oral cavity for hours after tooth brushing. Moreover, fluoride has the ability to inhibit the metabolism of bacteria, thus decreasing bacterial biofilm [2, 3]. Despite all these benefits of fluoride, there are some concerns about excessive intake of fluoride. It has been reported that ingestion of a large amount of fluoride containing dentifrice during tooth brushing in young children could result in permanent teeth fluorosis manifested as white or brown discolorations on the enamel surface [4–6].

Nevertheless, there are other potential sources of fluoride such as fluoridated water and milk as well as fluoride supplements that could also lead to chronic fluoride toxicity if ingested in large quantities [5]. Recent studies have drawn a great interest in incorporating herbal products to dentifrices owing to their anti-inflammatory, anti-microbial, sedative and analgesic activities as well as less toxicity, accessibility, and affordability [7, 8].

Ginger—also known as *Zingiber officinalis*—is one of the most commonly used dietary condiments in the world. It has been used for thousands of years for the treatment of numerous illness, such as common cold, arthritis, nausea, migraines, and hypertension. Ginger is considered a natural antibacterial, anti-inflammatory, and analgesic product. It is commonly used in the treatment of oral thrush and to relieve toothache. Over the last few years, interest in ginger products have increased markedly as a valid preventive or therapeutic agent [9, 10].

Ashwagandha—also known as *Withania somnifera*—is an Indian plant that has been used commonly in traditional medicine to improve the brain functions, enhance nervous system and to relieve stress. In addition, Ashwagandha exhibits potent anti-bacterial, anti-oxidant, anti-diabetic, antitumor, anti-inflammatory and anti-arthritic properties [11, 12].

Maca—also known as *Lepidium meyenii walp*—has been used for centuries in the Andes as a traditional medicine. An increasing interest in Maca products has been observed recently owing to its various therapeutic properties such as its ability to relieve rheumatism and respiratory disorders, and its effectiveness in treating anemia, leukemia, AIDS, and cancer. It also regulates hormonal secretion, stimulates metabolism, possess antidepressant activity and improves memory [13, 14].

The remineralization capacity of Ginger, Ashwaghanda and Maca showed promising results when evaluated in mouthwash [15]. Moreover, Ginger and Ashwaganda were efficient in occluding opened dentinal tubules and managing dentin hypersensitivity, however they showed inferior results compared to fluoride after acid attack [15]. To the best of our knowledge, the effect of Ginger, Maca and Ashwaghanda on enamel mineral deposition and enamel surface morphology, and microhardness are still lacking in literature. Accordingly, the aim of this study was to evaluate and compare the remineralization potential of experimentally prepared Ginger, Ashwaghanda and Maca herbal dentifrices versus commercially available fluoride containing dentifrice, and their effect on enamel surface microhardness.

The null hypothesis states that there would be no statistically significant difference between the experimentally prepared Ginger, Ashwagandha and Maca herbal based dentifrices and the commercially available fluoridated dentifrice regarding remineralization potential and enamel surface microhardness.

## Materials and methods

### Materials

Materials used for preparation of the experimental herbal dentifrices: Ginger, Ashwagandha and Maca were purchased from Egyptian local markets. Extra pure Tween 80 was purchased from (Loba-Chemie, India). Methanol, for liquid chromatography was purchased from (Millipore Merck, Germany). Lecithin was purchased from (Neogen, USA) and sodium deoxycholate monohydrate was purchased from (MP Biomedicals, USA). Whereas, Carbopol 940 was purchased from (Loba-Chemie, India). Commercially available fluoride containing dentifrice; (Signal cavity fighter, Unilever, England).

### Methodology

Study was accepted by the committee of research ethics, faculty of dentistry, Ain Shams university with approval number FDASU-Rec ER102308.

#### Preparation of Ginger, Ashwaghanda and Maca extracts

##### Extraction process

**Fig. 1:**
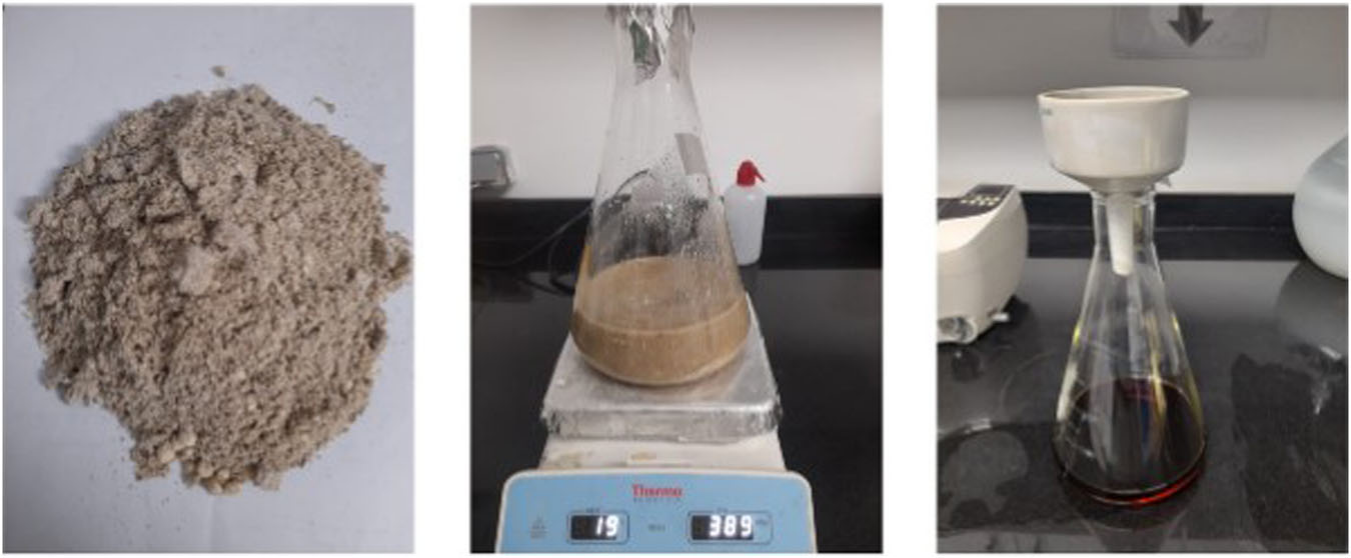
Extraction process of the herbal product.

10 g of dried Ginger, Ashwagandha and Maca powder were grinded and each is added to 100 mL of distilled water. The three formed solutions were mixed using a magnetic stirrer for 12 h at 80 °C and filtered twice through a paper filter as shown in Fig. 1. The solvent was then evaporated using hot plate magnetic stirrer at 90 °C overnight to obtain the final herbal extracts powder. In order to improve the topical delivery of the herbal extracts from the dentifrice, a lipid drug carrier (liposome) was formulated.

##### Preparation of herbal extract/liposome complex

Three beakers were used, each contained 180 mg of Lecithin, 12 mg of tween 80 and 8 mg of sodium deoxycholate monohydrate dissolved in 100 ml of methanol. Methanol was further evaporated by rotary evaporation (D-Lab with Water bath, USA). Then a solution of 100 ml of phosphate buffer saline solution (pH 7.4) and 40 mg of each herbal extract was prepared and added to each beaker. The mixtures were then centrifuged at 150 rpm at 45 °C, followed by sonication for 30 min in a probe sonicator to obtain Ginger, Ashwaghanda and Maca liposome complex [16–19].

##### Preparation of Ginger, Ashwaghanda and Maca dentifrices

0.25 gm of each prepared herbal/liposome complex was dissolved in 50 ml distilled water and mixed using a magnetic stirrer. Then 0.5 gm of Carbopol940 (gelling agent) was added to each prepared solution to form the dentifrice gel.

#### Characterization of the herbal extracts and tested dentifrices

Size and morphology of the prepared Ginger, Ashwagandha and Maca were assessed by transmission electron microscope (TEM) (JEM-2100, JEOL.USA) at an accelerating voltage of 200 kV. Dynamic light scattering (DLS) (Nano ZS90 analyzer, Malvern Instruments, Malvern.UK) was used to measure the zeta potential and particle size (hydrodynamic diameter D_*h*_) using distilled water as a diluent for the samples. Calcium (Ca), phosphorus (P) and fluoride (F) ions release were determined using an inductively coupled plasma optical emission spectrometer, ICP-OES 5800 (Spectro Analytical instruments, Agilent, USA). The pH of the four tested dentifrices was measured using a digital pH meter (Hanna Instruments, Inc., USA). 5 g of each dentifrice were weighed on a precision scale, followed by dilution and mixing with 15 ml of distilled water [20]. The pH meter was calibrated between readings with a neutral solution (pH = 7).

#### Preparation of artificial saliva

Artificial saliva was prepared by mixing CaCl_2_.H_2_O (0.795 g), NaH_2_PO_4_. H_2_O (0.690 g), KCl (0.400 g), NaCl (0.400 g), and Na_2_S.9H_2_O (0.005 g) in 1000 mL of deionized water. The pH was adjusted by adding 1 M of NaOH to maintain a neutral pH of 7.0 [21].

#### Preparation of demineralizing agent

The demineralizing solution was prepared and composed of CaCl_2_ (2.2 mM), KH_2_PO_4_ (2.2 mM), 0.05 M of acetic acid, and 1 M of KOH to maintain a pH of 4.4 [22].

#### Specimen’s preparation and grouping

The sample size was calculated depending on previous study as a reference [8], using power and sample size calculation software (version 3.1.9.7). Sample size was calculated to be 13 for each experimental group (*n* = 13) with power value of 80% and type I error probability of 0.05.

**Fig. 2:**
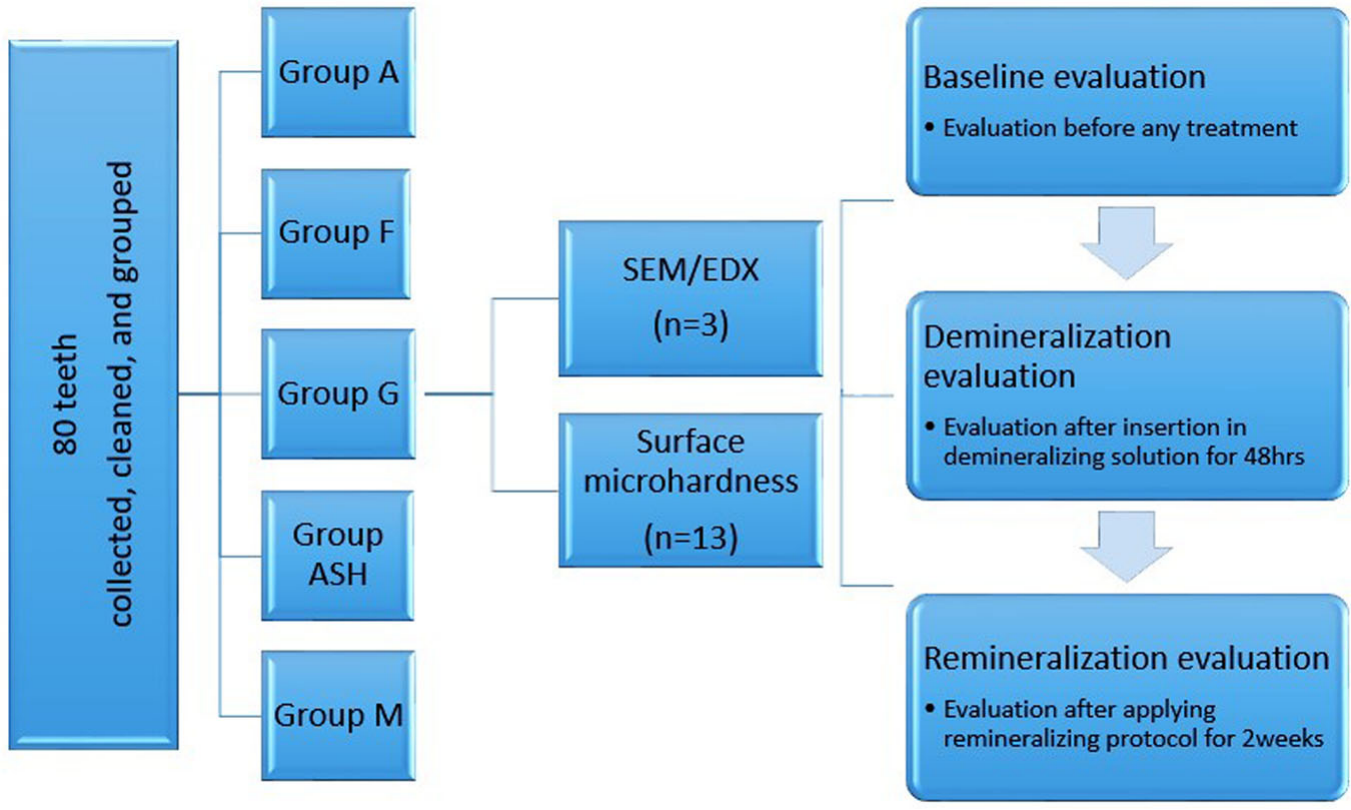
Experimental design of the study.

A total of eighty human molars and premolars extracted for therapeutic causes were collected and divided randomly into five groups (*n* = 13 for microhardness testing and *n* = 3 for SEM-EDX analysis) according to the remineralization protocol as follows (Fig. 2):

- **Group A:** Stored in artificial saliva (negative control).
- **Group F:** Treated with commercially available fluoridated dentifrice (Signal cavity fighter, Unilever, England) (positive control).
- **Group G:** Treated with experimentally prepared Ginger dentifrice.
- **Group ASH:** Treated with experimentally prepared Ashwaghanda dentifrice.
- **Group M:** Treated with experimentally prepared Maca dentifrice.

The collected teeth were devoid of any fractures or cracks, any visible discoloration, caries or white spot lesions, developmental abnormalities, endodontic therapy or restorations. The collected teeth were thoroughly washed, and any calculus was gently removed with manual scalers. The specimens were kept in normal saline until usage to avoid dehydration.

The crowns of the collected teeth were then separated from the roots by cutting them at the level of the cemento-enamel junction using a low-speed diamond saw (Isomet 4000, Buehler, USA). Each crown was then embedded in self-cured acrylic resin block with the buccal surface facing upwards. The crown surfaces were then coated with an acid resistant nail varnish leaving a 4 × 4 mm window in the center of the buccal surface.

### Testing

#### Enamel surface morphology and mineral content evaluation

Three specimens from each group (*n* = 3) were examined using scanning electron microscope (SEM) to evaluate enamel surface morphology with an accelerating voltage of 20 kV. Energy dispersive X-ray (EDX) spectroscopy analyzer software were used to measure the atomic percentages of calcium, phosphorus and carbon elements in enamel to indicate changes in the mineral contents [23].

Enamel surface morphology and mineral content of the specimens were evaluated at baseline (BL), after demineralization (DM) and after remineralization (RM). The calcium and phosphorus atomic percentages after remineralization were then converted into Ca/P ratios for each group from the obtained data, as they indicate alterations in the inorganic components of hydroxyapatite [24].

#### Demineralization (DM) protocol

Specimens were subjected to a demineralization process after BL evaluation, where each specimen was immersed in 10 ml of the prepared demineralized solution for 48 hours at 37 °C. The demineralization solution was changed daily to avoid depletion of the solution. All teeth in all groups were subjected to the same demineralizing solution, for the same period of time for standardization purpose. After DM, the specimens were washed with distilled water, dried and then evaluated.

#### Remineralization (RM) protocol

Specimens of all groups were treated with the allocated protocol after DM evaluation as mentioned above. For groups F, G, ASH and M; an applicator brush was used to apply the dentifrice for 2 min, twice a day for 2 weeks. After each brushing, the specimens were rinsed with distilled water for 30 s, kept in artificial saliva and stored at room temperature [2]. While specimens of group A (negative control) were stored at room temperature in artificial saliva.

#### Enamel surface micro-hardness (SMH) evaluation

Thirteen specimens from each group (*n* = 13) were tested for surface micro-hardness using Vickers micro-hardness tester with digital display (Model HVS-50, Laizhou Huayin Testing Instrument Co., Ltd. China). A load of 200 g was applied to the surface of each specimen by the Vickers diamond indenter for 20 seconds. Three indentations were made away from the margins of the specimens and not closer than 0.5 mm to the adjacent indentation. A built-in scaled microscope was used to measure the lengths of the indentations diagonals. SMH was calculated according to the following equation [25].

$${{{\rm{HV}}}}=1.854{{{\rm{P}}}}/{{{\rm{d}}}}2$$

Where HV is Vickers hardness in KgF/mm^2^, P is the load in Kg and d is the length of the diagonals in mm.

Enamel surface microhardness of the specimens was evaluated at BL, after DM and after RM. The extent of remineralization was calculated as percentage of surface microhardness recovery (SMHR) as follows [26]:

$$[({{{\bf{SMH}}}}\; {{{\bf{after}}}}\; {{{\bf{RM}}}}-{{{\bf{SMH}}}}\; {{{\bf{after}}}}\; {{{\bf{DM}}}})/({{{\bf{SMH}}}}\; {{{\bf{at}}}}\; {{{\bf{BL}}}}-{{{\bf{SMH}}}}\; {{{\bf{after}}}}\; {{{\bf{DM}}}})]\times {{{\bf{100}}}}$$

### Statistical analysis

Mean values and standard deviation for each group were tested for normality using Shapiro-Wilk test and Kolmogorov-Smirnov tests which revealed normal data. One-way ANOVA followed by Tukey’s post hoc test were used in this study to analyze EDX and SMH results. The significance level was set at *P* ≤ 0.05. Statistical analysis was performed with (IBM SPSS Statistics 20) for Windows.

## Results

### Transmission electron microscope (TEM) analysis results

**Fig. 3:**
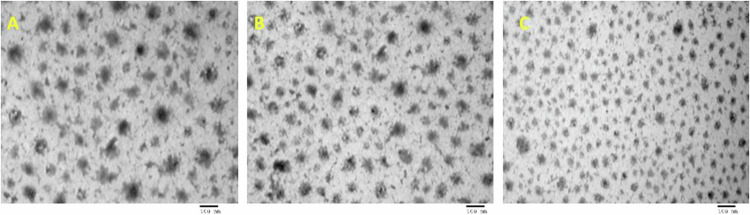
TEM analysis of the prepared herbal products at magnification (10000X). **A** Ginger/liposomal complex, **B** Ashwaganda/liposomal complex, and **C** Maca/liposomal complex.

**Fig. 4:**
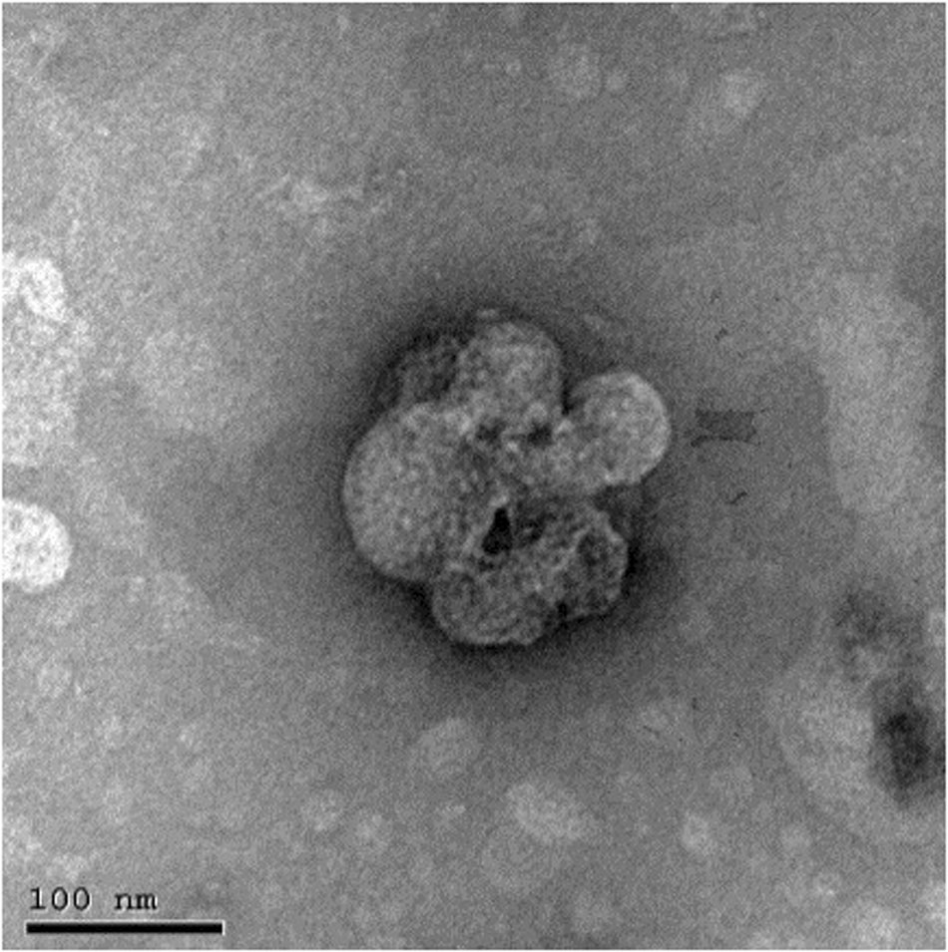
TEM analysis at scale bar of 100 nm of Ginger/liposomal complex.

TEM analysis at magnification (10000×) (Fig. 3) revealed the morphology of the prepared herbal products which appeared as nano-sized nodules. TEM analysis at scale bar 100 nm (Fig. 4) revealed ginger/ liposomal drug carrier with size of approximately 60 nm.

### Dynamic light scattering (DLS) results

DLS results (Table 1) revealed that Maca showed the lowest zeta potential value of (−30.6 mV) and also showed the smallest particle size of (106.8 nm). However, Ginger and Ashwaganda showed zeta potential of (−26.1 and −27.5 respectively) and particle size of (242.3 and 241.6 respectively).

**Table 1:** Zeta potential values (mV) and particle size (D_*h*_) (nm) of Ginger, Ashwaganda and Maca extracts.

Specimen	Zeta potential (mV)	D_*h*_ (nm)
**Ginger**	−26.1	242.3
**Ashwaganda**	−27.5	241.6
**Maca**	−30.6	106.8

### Calcium, phosphorus and fluoride ion release results

ICP results (Table 2) showed that the highest Ca ion release was attributed to Maca (71892.49 ppm), followed by Ashwagandha (34251.65 ppm ppm), then Ginger (15834.92 ppm). While the highest phosphorus release was attributed to Ashwagandha (12736.53 ppm), followed by Maca (3758.88 ppm), then Ginger (826.71 ppm). However, the highest fluoride release was associated with Ginger (24.21 ppm), followed by Maca (11.21 ppm), then Ashwagandha (8.12 ppm).

**Table 2:** Calcium and phosphorus release (ppm) from Ginger, Ashwaganda and Maca herbal products performed by ICP.

Specimen	Calcium	Phosphorus	Fluoride
**Ginger**	15834.92	826.71	24.21
**Ashwaganda**	34251.65	12736.53	8.12
**Maca**	71892.49	3758.88	11.21

### pH analysis results

pH results (Table 3) showed that Ginger dentifrice exhibited the highest pH value of 7.2, followed by signal dentifrice (pH = 6.8), then Maca (pH = 6.5), whereas Ashwaganda dentifrice showed the lowest pH value (pH = 5.4).

**Table 3:** pH values of the tested dentifrices.

Dentifrice Type	pH values
**Signal Dentifrice**	**6.8**
**Ginger Dentifrice**	**7.2**
**Ashwagandha Dentifrice**	**5.4**
**Maca Dentifrice**	**6.5**

### Scanning electron microscope (SEM) analysis results

**Fig. 5:**
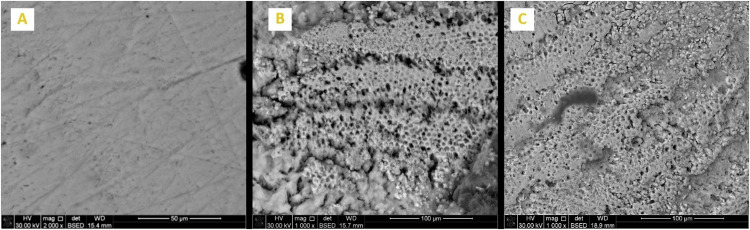
SEM analysis of group A (negative control group). **A** At baseline (at 2000X), **B** after DM (at 1000X), **C** after RM (at 1000X).

**Fig. 6:**
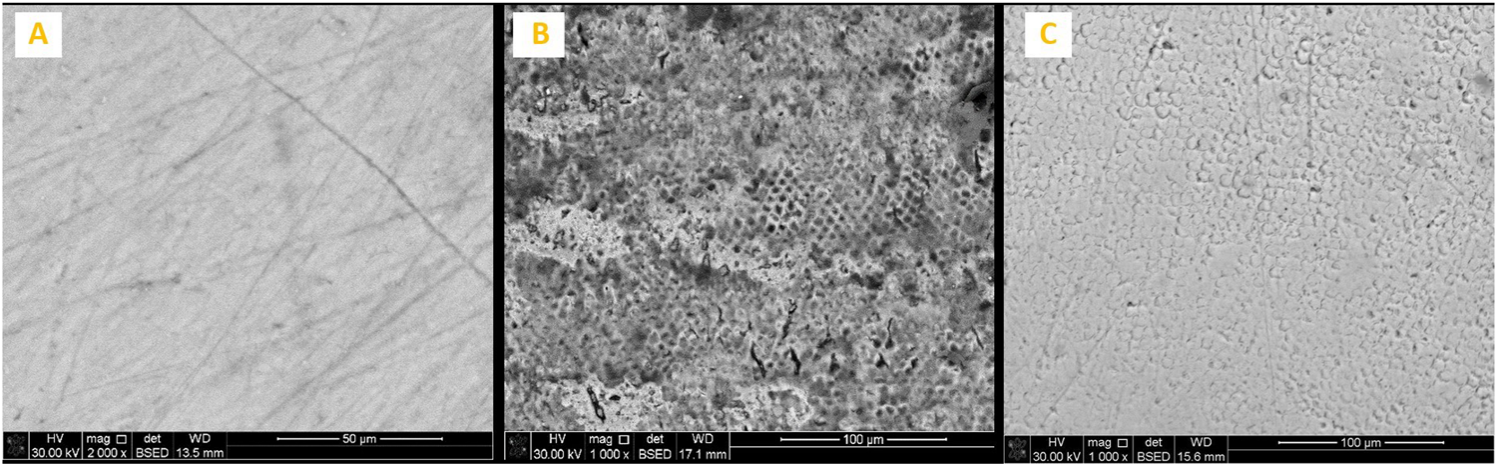
SEM analysis of Group F (positive control). **A** At baseline (at 2000X), **B** after DM (at 1000X), **C** after RM (at 1000X).

**Fig. 7:**
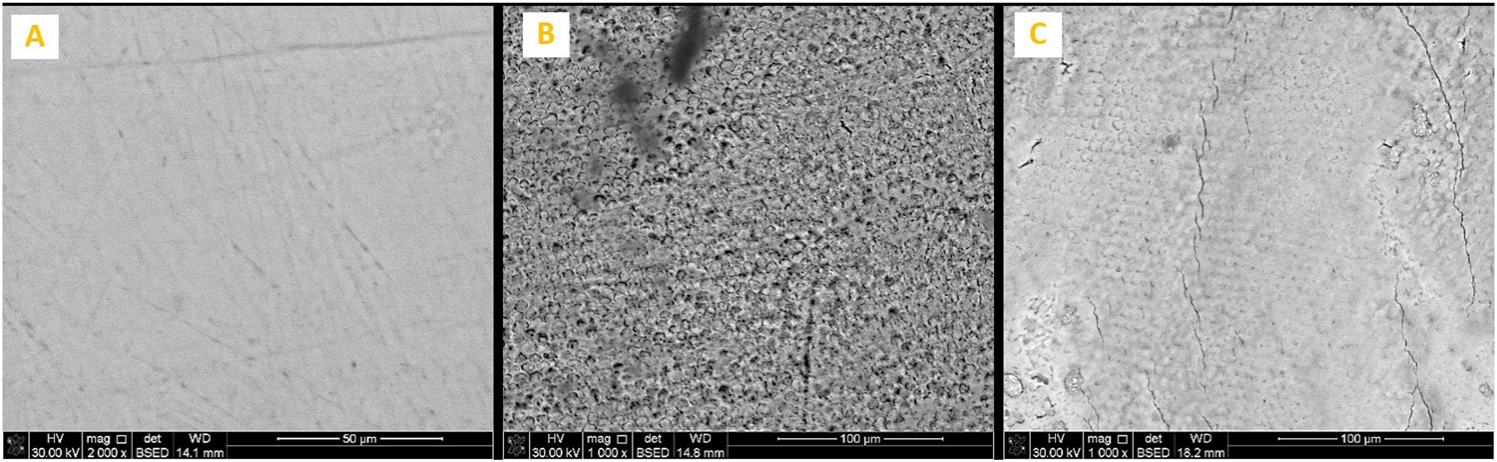
SEM analysis of Group G. **A** At baseline (at 2000X), **B** after DM (at 1000X), **C** after RM (at 1000X).

**Fig. 8:**
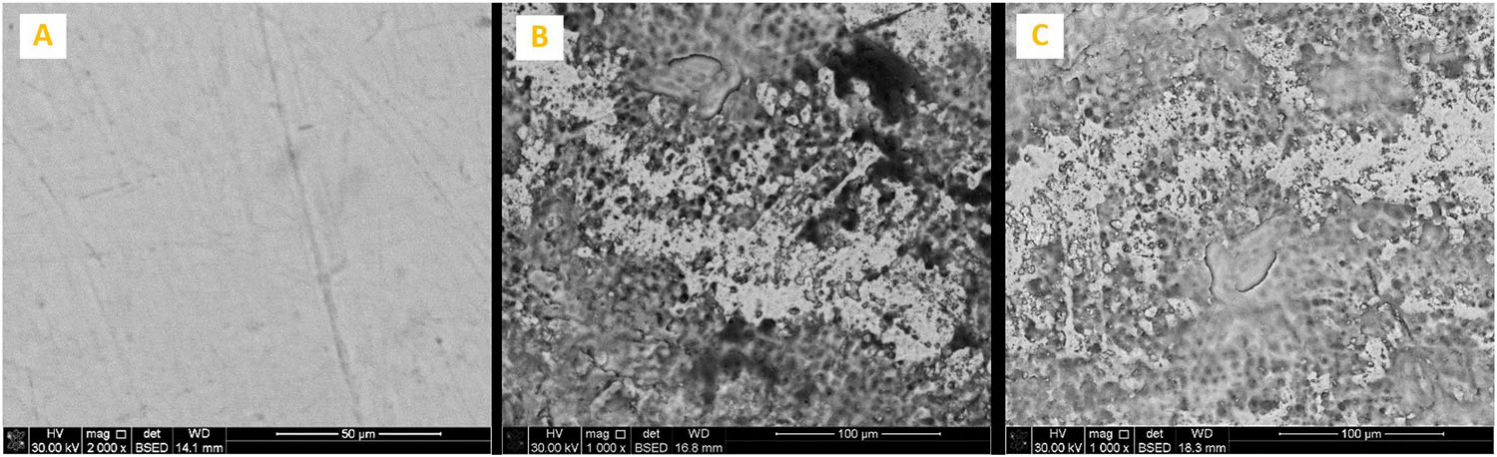
SEM analysis of Group ASH. **A** At baseline (at 2000X), **B** after DM (at 1000X), **C** after RM (at 1000X).

**Fig. 9:**
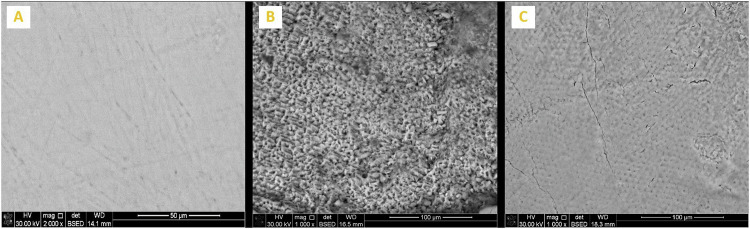
SEM analysis of Group M. **A** at baseline (at 2000X), **B** after DM (at 1000X), **C** after RM (at 1000X).

Enamel surface morphology of all tested specimens at BL exhibited a generalized smooth and flat surface with homogeneous appearance with visible scratches that could be caused by polishing (Figs. 5A, 6A, 7A, 8A and 9A). Whereas, after DM, the enamel surfaces of all tested specimens (Figs. 5B, 6B, 7B, 8B and 9B) showed alteration in the integrity of enamel surface and disturbance in prismatic structure with inter-prismatic gaps and porosity in the form of fish scales or honeycomb prismatic pattern destruction [27]. After RM, groups F, G and M showed improvement in surface morphology with relatively smooth enamel surface compared to their DM images (Figs. 6C, 7C and 9C). However, group ASH exhibited rough areas with detected disturbances in the prismatic structure (Fig. 8C). There was no morphological difference between DM and RM images of group A (negative control) (Fig. 5).

### EDX results

**Fig. 10:**
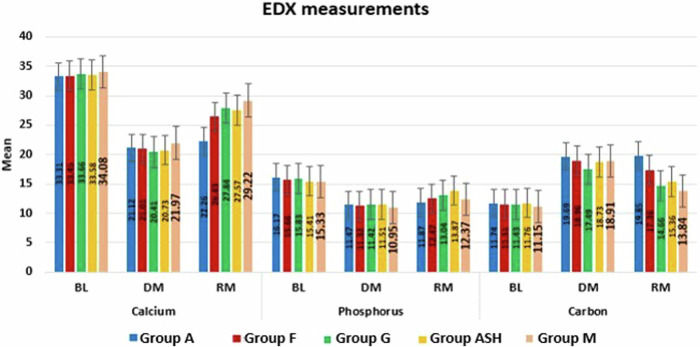
Bar chart showing calcium, phosphorous, and carbon at BL, after DM and after RM in all groups.

EDX results (Table 4 and Fig. 10) revealed significant decrease in Ca and P mean values and significant increase in C mean values in all tested groups from BL to after DM. However, groups F, G, ASH and M showed significant increase in Ca and P mean values and significant decrease in C mean values after RM. The results also showed insignificant difference after RM in Ca, P and C mean values of group A.

**Table 4:** Mean (M) and standard deviation (SD) of calcium, phosphorous, and carbon at baseline (BL), at demineralization (DM) and after remineralization (RM) of all tested groups.

Groups		Group A		Group F		Group G		Group ASH		Group M	
		M	SD	M	SD	M	SD	M	SD	M	SD
**Calcium**	BL	33.31^**a**^	0.61	33.45^**a**^	0.78	33.66^**a**^	0.73	33.58^**a**^	0.84	34.08^**a**^	.59
	DM	21.12^**b**^	0.78	21.01^**b**^	1.09	20.41^**b**^	1.24	20.73^**b**^	1.32	21.97^**b**^	1.30
	RM	22.26^**b**^	2.20	26.43^**c**^	1.03	27.84^**c**^	1.31	27.57^**c**^	0.70	29.22^**c**^	.80
	*P* value	0.0001*		0.0001*		0.0001*		0.0001*		0.0001*	
**Phosphorus**	BL	16.17^**a**^	0.74	15.66^**a**^	0.97	15.83^**a**^	0.70	15.41^**a**^	1.28	15.33^**a**^	0.71
	DM	11.47^**b**^	0.83	11.32^**b**^	0.93	11.42^**b**^	0.82	11.51^**b**^	0.89	10.95^**b**^	1.07
	RM	11.87^**b**^	0.87	12.47^**c**^	0.76	13.04^**c**^	0.94	13.87^**c**^	1.03	12.37^**c**^	1.31
	*P* value	0.0001*		0.0001*		0.0001*		0.0001*		0.0001*	
**Carbon**	BL	11.74^**a**^	0.88	11.51^**a**^	0.74	11.43^**a**^	0.82	11.76^**a**^	0.83	11.15^**a**^	.65
	DM	19.69^**b**^	0.62	18.96^**b**^	0.72	17.49^**b**^	0.80	18.73^**b**^	0.69	18.92^**b**^	.45
	RM	19.85^**b**^	0.91	17.36^**c**^	0.65	14.66^**c**^	0.76	15.36^**c**^	0.82	13.88^**c**^	1.07
	*P* value	0.0001*		0.0001*		0.0001*		0.0001*		0.0001*	

Means with different superscript letters per column indicate significant difference at *P* < 0.05.

**Fig. 11:**
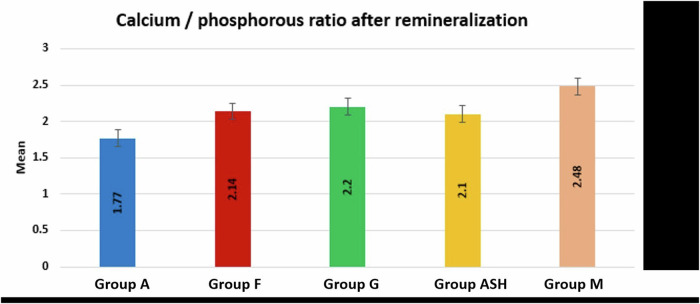
Bar chart showing calcium/phosphorous ratio after remineralization in all groups.

Ca/P ratio results (Table 5 and Fig. 11) showed that group M (2.48 ± 0.11) revealed a significantly higher Ca/P mean value compared to all other tested groups, while group A (1.77 ± 0.10) revealed a significantly lower Ca/P mean value compared to all other tested groups (*p* < 0.001). However, the difference between F, G and ASH groups was insignificant.

**Table 5:** Mean (M) and standard deviation (SD) values of calcium/phosphorous ratio after remineralization of all tested groups.

Groups	M	SD	*P* value
**Group A**	1.77^**a**^	0.10	<0.00001*
**Group F**	2.14^**b**^	0.19	
**Group G**	2.20^**b**^	0.26	
**Group ASH**	2.10^**b**^	0.21	
**Group M**	2.48^**c**^	0.11	

Means with different superscript letters per column indicate significant difference at *P* < 0.05.

### Surface microhardness (SMH) results

**Fig. 12:**
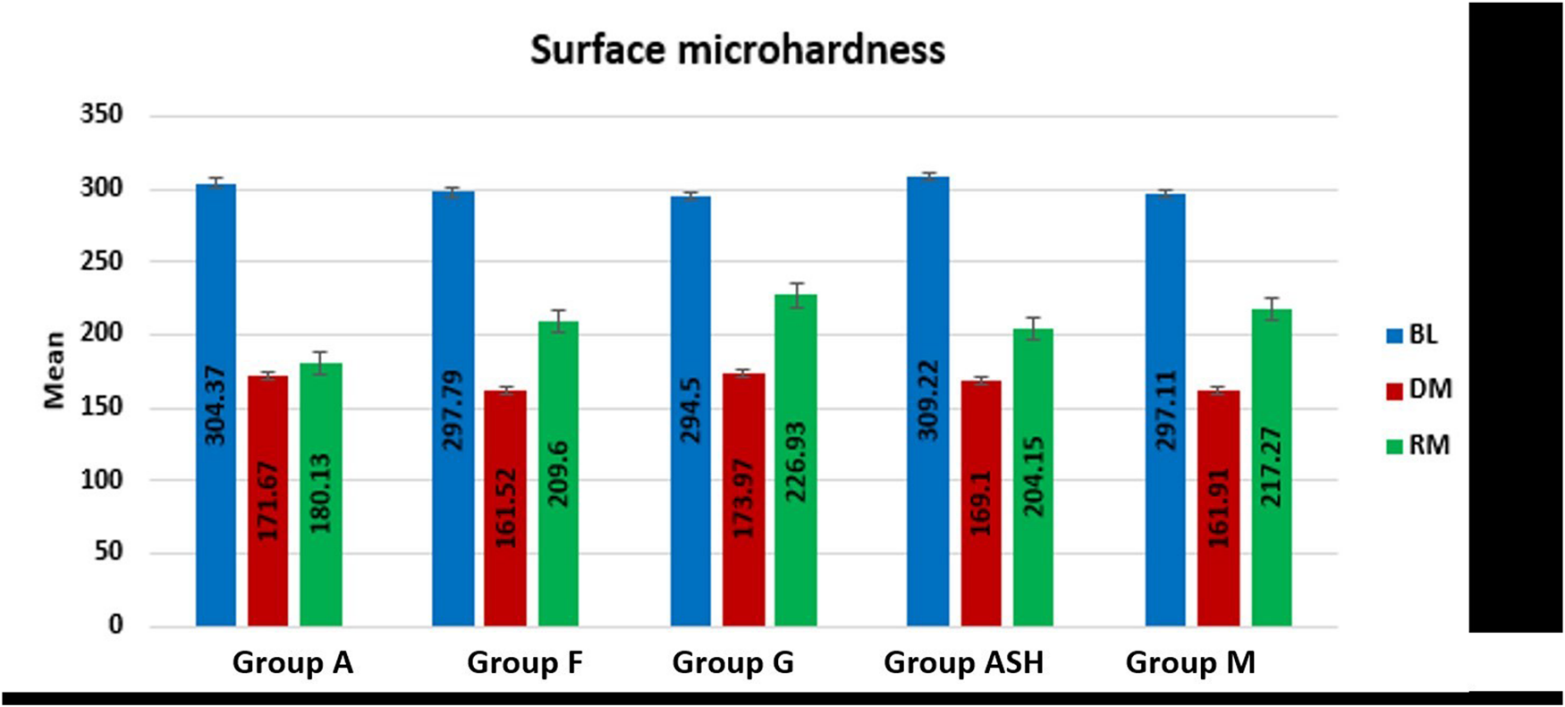
Bar chart showing surface microhardness at BL, after DM and after RM in all groups.

The results (Table 6 and Fig. 12) revealed significant decrease in SMH mean values in all tested groups from BL to after DM. However, groups F, G, ASH and M showed significant increase in SMH values after RM. The results also showed insignificant difference in SMH value after RM in group A.

**Table 6:** Mean (M) and standard deviation (SD) values of surface microhardness (SMH) at baseline (BL), at demineralization (DM) and after remineralization (RM) of all tested groups.

Surface microhardness	Group A		Group F		Group G		Group ASH		Group M	
	M	SD	M	SD	M	SD	M	SD	M	SD
**BL**	304.37^**a**^	13.38	297.79^**a**^	29.11	294.50^**a**^	28.85	309.22^**a**^	16.45	297.11^**a**^	16.37
**DM**	171.67^**b**^	9.75	161.52^**b**^	11.57	173.97^**b**^	36.28	169.10^**b**^	8.89	161.91^**b**^	11.53
**RM**	180.13^**b**^	20.48	209.60^**c**^	9.98	226.93^**c**^	28.56	204.15^**c**^	26.99	217.27^**c**^	16.50
***P*** **value**	0.00001*		0.00001*		0.00001*		0.00001*		0.00001*	

Means with different superscript letters per column indicate significant difference at *P* < 0.05.

### Surface micro-hardness recovery (SMHR) results

**Fig. 13:**
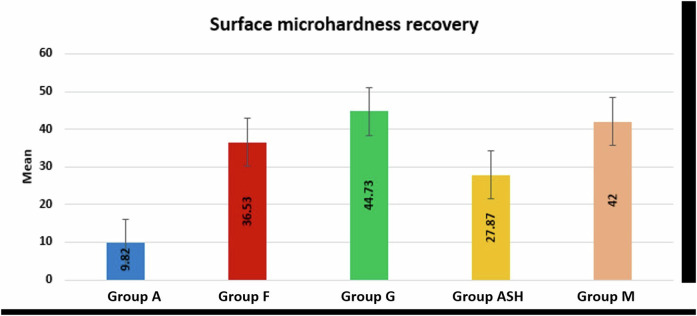
Bar chart showing surface microhardness recovery in all groups.

SMHR results (Table 7 and Fig. 13) showed that group G (44.73 ± 11.69) revealed a significantly higher SMHR mean value compared to group ASH (27.87 ± 10.25) and group A (9.82 ± 4.03) at *p*-value < 0.001, but with no statistical significant difference with groups F and M.

**Table 7:** Mean (M) and standard deviation (SD) values of surface microhardness recovery of all tested groups.

Groups	M	SD	*P* value
**Group A**	9.82^**a**^	4.03	<0.00001*
**Group F**	36.53^**bc**^	7.69	
**Group G**	44.73^**b**^	11.69	
**Group ASH**	27.87^**c**^	10.25	
**Group M**	42^**bc**^	9.96	

Means with different superscript letters per column indicate significant difference at *P* < 0.05.

## Discussion

Fluoride has been used in various oral hygiene products owing to its beneficial effects on reducing the prevalence of dental caries. However, ingestion of high levels of fluoride in young age may result in dental fluorosis [4–6]. The use of natural compounds in the composition of oral hygiene products has proved to be a good cost-effective alternative for chemical ingredients, as they provide a more sustainable environment owing to their great therapeutic benefits [28, 29]. In this study, 0.5% of Ginger, Ashwaganda and Maca herbs were used to prepare three experimental herbal based dentifrices. Solvent extraction methodology was used to prepare the three herbal products, as it is the most commonly used method because it is cost effective and convenient [30]. Distilled water was used as solvent in the extraction process, as it is the most commonly used solvent owing to its high extraction efficiency, low cost, biocompatibility, and non-inflammability [31, 32]. Lipid drug carriers named as (liposomes) were formulated to improve the topical delivery of the herbal extracts, enhance their therapeutic effects and improve their stability and solubility [16, 33]. A concentration of (0.5%) of herbal extract was used in this study, as according to AL-Azawi et al. [34], there is a maximum amount of calcium ions that can be deposited on the enamel surface and above this amount, other constituent elements from the herbal extracts will increase as well, which may substitute calcium ions from enamel surface decreasing the remineralization efficacy [34]. In this study, the tested dentifrices were applied on enamel specimens twice a day to simulate the normal recommended daily use of prophylaxis dentifrices.

The TEM analysis of the prepared herbal products confirmed the preparation of the herbal products in nano-size. Zeta potential is often used as an indicator for stability of nanoparticles, where values more positive than +30 mV or more negative than −30 mV indicate good stability against agglomeration due to electrostatic repulsion between the particles [35]. According to the results of our study, it could be suggested that Maca (zeta potential = −31.2) exhibited better dispersion and stability against coalescence which was further indicated by their least particle size (D_*h*_ = 106 nm). ICP was used to assess the Ca, P and F release from the prepared herbal products, as it is an accurate methodology for the analysis of leached ions in trace levels. The results showed that Maca exhibited the highest Ca ion release (71892.49ppm), whereas, the Ashwagandha exhibited the highest P ion release (12736.53 ppm). It was reported that Ashwaganda contains high amount of phosphorus in most of the plant parts, including leaves, flowers, fruits and seeds [36]. However, the highest fluoride release was attributed to Ginger (24.21 ppm).

The pH value of dentifrices is related to the effectiveness and stability, as well as it plays an important role in remineralization ability, as it was emphasized that remineralization occurs under near-neutral physiological pH conditions [1, 37, 38]. The pH results of this study showed that all tested dentifrices were within the safe pH range (4.5-10.5). Where, Ginger, fluoride and Maca containing dentifrices exhibited near neutral pH (7.2, 6.8 and 6.5 respectively). while Ashwaganda containing dentifrice exhibited slightly acidic pH of 5.4

In this study, SEM/EDX analysis was performed to evaluate the enamel surface morphology and quantitavely measure changes in the mineral contents at baseline, after demineralization and after remineralization [23]. Surface microhardness testing has been utilized as it provides a relatively simple, non-destructive, reliable and rapid method in demineralization and remineralization studies. Microhardness measurement is appropriate for materials with fine microstructure and prone to cracking like enamel [39]. SEM images of all groups after demineralization showed irregular porous surface caused by increasing in the inter-crystalline spaces, as well as obvious destruction in the prismatic patterns confirming the success of the demineralization protocol [40]. This was in agreement with the EDX results, that exhibited statistically significant decrease in the mineral contents (Ca and P) after immersion in the demineralizing solution that caused dissolution of Ca and P from apatite crystals as well as microstructural damage. The results also showed significant increase in carbon content in all tested groups, as during demineralization higher amounts of carbonates substituted minerals in hydroxyapatite lattice, resulting in poor crystalline and porous enamel [40–42]. The results of SEM/EDX were parallel to the SMH results that showed an overall significant decrease in microhardness in demineralized enamel surfaces in all groups as a result of minerals loss.

After remineralization, the SEM images of groups F, G, ASH and M showed relatively smooth surfaces with obvious obliteration of the enamel micro-porosities. This could indicate the ability of Signal dentifrice, as well as the experimentally prepared Ginger, Ashwaganda and Maca dentifrices to substantially deposit Ca and P forming new hydroxyapatite crystals, sealing and filling up the previously exposed enamel defects which accordingly improve the surface texture as well as microhardness. Nevertheless, SEM results showed that the quality of enamel surface of ASH group (fig8) exhibited relatively porous and inhomogenous surface denoting disturbances in the prismatic structure indicating poor remineralization. This was in accordance with SMHR results, where ASH group showed significantly lower SMHR value compared to Ginger group (*p*-value < 0.001). This could be attributed to the slight acidity of Ashwaganda dentifrice (pH 5.4) which could result in dissolution and subsequent weakening of hydroxyapatite crystals in enamel [43]. Besides, Ashwaganda nanoparticles are composed mainly of highly reactive low-molecular weight nitrogen-containing compounds called “alkaloids” [12]. Alkaloids have basic properties and they form crystalline salts in acidic medium [44]. It could be suggested that the acidity of Ashwaganda dentifrice could lead to the precipitation of some of these crystalline salts into enamel micro porosities which could decrease the remineralization process. This could also explain the SEM findings of ASH group.

Ginger exhibited higher amount of fluoride ions release compared to Ashwaganda, as shown by ICP (Table 2). Fluoride plays a pivotal role in remineralization by encouraging Ca^2+^ to attach to the tooth surface. Gocmen et al. reported that the remineralization efficiency of Ginger is attributed to its high fluoride release [44]. However, the exact remineralization effect of Ginger is unknown, but other studies revealed its efficiency in tooth remineralization [15, 26, 34, 45, 46]. Moreover, the near-neutral pH of Ginger containing dentifrice (pH = 7.2) could favor the deposition of high amounts of calcium and phosphorus ions into the pores of the demineralized enamel surface as reported in previous studies [1, 47]. The results of this study was in accordance with Abd ElAziz (2024) et al. [15], who compared Ginger, Maca and Ashwaganda mouthwashes to fluoride containing mouthwash. They observed high remineralization capacity of the tested herbal products. However, the results of their study showed that the tested herbal mouthwashes exhibited statistically significant higher remineralizing capacity compared to fluoride mouthwash. This disagreed with our findings, that showed no statistically significant difference between fluoride dentifrice and Ginger, Maca or Ashwaganda dentifrices. This discrepancy could be attributed to the difference in herbal preparation methodology as well as the difference in the composition between dentifrice gel and mouthwash which could affect pH, particle size, zeta potential of the prepared formulations and hence the remineralization kinetics. Moreover, in Abd ElAziz et al. study, DIAGNOdent pen was used to evaluate the remineralization efficiency. DIAGNOdent utilizes a different methodology which depends on the absorption of diode laser by the organic and inorganic content of the tooth structure, and it was believed that this could probably show different results than Vickers surface microhardness test, especially that DIAGNOdent exhibits relatively low specificity for enamel lesions owing to its lower organic content [48]. Abd ElAziz (2024) et al. [15], conducted another study to evaluate the efficiency of Ginger and Ashwaganda to treat dentin hypersensitivity. In their study, fluoride containing Sensodyne dentifrice exhibited significantly better results compared to Ginger and Ashwaganda after acid attack. They explained that the presence of fluoride phosphate complex in the composition of Sensodyne dentifrice increased its bioactivity and supported efficient dentinal tubules occlusion.

The results of this study also showed that Maca group exhibited the highest Ca/P ratio compared to all other tested groups. This could be attributed to their highest calcium ion release (71892.49 ppm) as shown in ICP results (Table 2). Moreover, it was concluded before from DLS results that Maca exhibited the smallest nanoparticle size (D_*h*_ = 106 nm) and the best dispersion owing to their zeta potential (−30.6 mV). This could allow them to effectively fill the microscopic porosity of enamel surface enhancing mineral deposition.

The inferior SEM/EDX and SMH results of group A (negative control), indicated lack of surface remineralization, owing to the use of artificial saliva that has limited ions concentration available for remineralization.

### Limitations of the study

This is an in vitro study that evaluated the effect of different natural demineralizing agents on hydroxyapatite crystals of tooth structure outside the oral cavity. However, in vivo conditions are different and the effects measured in vitro are not necessarily replicated in vivo. So, in vivo studies should be taken in consideration in further researches. Different concentrations and combinations of the tested remineralizing agents need also to be examined. Moreover, other properties including microbiological, biocompatibility, viscosity and spread-ability are recommended to be considered in further studies.

## Conclusions

Ginger and Maca containing dentifrices showed comparable results to commercially available fluoride containing dentifrice, and it could be concluded that Ginger and Maca herbal products are promising remineralizing agents. However, more research is needed before considering these as a substitution for fluoride.

## Data Availability

The data that support the findings of the study are available on request from the corresponding author.
